# Supplementary material for: Mechanisms governing the pioneering and redistribution capabilities of the non-classical pioneer PU.1
Source: Nat Commun. 2020 Jan 21;11:402. doi: 10.1038/s41467-019-13960-2 (PMC6972792; doi:10.1038/s41467-019-13960-2)
Supplement: Supplementary file 7 — Source data [file 41467_2019_13960_MOESM7_ESM.zip › Source_Data/FigureS2/FigureS2B_MotifScanOutput/KmeansCluster9/homerResults/motif5.similar.html]

motif5

## Information for motif5

C
T
A
G
G
T
A
C
A
G
T
C
G
C
T
A
A
T
G
C
G
A
T
C
C
G
T
A
A
C
T
G
C
T
G
A
C
A
T
G
A
C
T
G
A
C
T
G
G
T
A
C
T
C
G
A
  
Reverse Opposite:  

A
G
C
T
C
A
T
G
G
T
A
C
A
G
T
C
G
T
A
C
A
G
C
T
A
G
T
C
A
C
G
T
C
T
A
G
A
T
C
G
C
G
A
T
A
C
T
G
C
A
T
G
G
A
T
C
  

|  |  |
| --- | --- |
| p-value: | 1e-91 |
| log p-value: | -2.112e+02 |
| Information Content per bp: | 1.709 |
| Number of Target Sequences with motif | 121.0 |
| Percentage of Target Sequences with motif | 5.32% |
| Number of Background Sequences with motif | 125.0 |
| Percentage of Background Sequences with motif | 0.27% |
| Average Position of motif in Targets | 105.8 +/- 50.7bp |
| Average Position of motif in Background | 100.8 +/- 62.5bp |
| Strand Bias (log2 ratio + to - strand density) | -0.1 |
| Multiplicity (# of sites on avg that occur together) | 1.02 |
| Motif File: | file (matrix) reverse opposite |

### Similar de novo motifs found

|  |  |  |  |  |  |  |  |
| --- | --- | --- | --- | --- | --- | --- | --- |
| Rank | Match Score | Redundant Motif | P-value | log P-value | % of Targets | % of Background | Motif file |
| 1 | 0.976 | A G C T C A T G G T A C A G T C G T A C A G C T G T A C A G C T C T A G T A C G C G A T A C T G A C T G | 1e-84 | -193.961382 | 6.28% | 0.57% | motif file (matrix) |
| 2 | 0.928 | C A T G G A T C G T A C G T A C G A C T A G T C G A C T C T G A T A C G G C A T A C T G A C T G | 1e-66 | -152.954915 | 5.18% | 0.51% | motif file (matrix) |
| 3 | 0.934 | A G T C C T G A T A G C A G C T C G T A A C T G C T A G C A T G A C T G C T A G G T A C | 1e-56 | -130.393276 | 6.20% | 1.01% | motif file (matrix) |
| 4 | 0.898 | A G T C C T G A T A G C G C A T C G T A A C T G C T A G C A T G A C T G C T A G | 1e-44 | -103.593035 | 6.06% | 1.26% | motif file (matrix) |
| 5 | 0.777 | A G C T C G T A A C T G C T G A C A T G A C T G A C T G A G T C C T A G | 1e-20 | -48.239118 | 6.33% | 2.49% | motif file (matrix) |
